# Possible role of nanocarriers in drug delivery against cervical cancer

**DOI:** 10.1080/20022727.2017.1335567

**Published:** 2017-07-07

**Authors:** Swati Gupta, Manish K. Gupta

**Affiliations:** a B. S. Anangpuria Institute of Pharmacy, Pt B. D. Sharma University of Health Sciences, Faridabad, India; b TERI-Deakin Nano Biotechnology Centre, The Energy and Resources Institute, Gurugram, India

**Keywords:** Cervical cancer, liposomes, nanoparticles, targeting, colloidal carriers, chemotherapy

## Abstract

**Introduction:** Cervical cancer is the second most common cancer and the largest cancer killer among women in most developing countries including India. Although, various drugs have been developed for cervical cancer, treatment with these drugs often results in a number of undesirable side effects, toxicity and multidrug resistance (MDR). Also, the outcomes for cervical cancer patients remain poor after surgery and chemo radiation.

**Methods:** A literature search (for drugs and delivery systems against cervical cancer) was performed on PubMed and through Google. The present review discuss about various methods including its current conventional treatment with special reference to recent advances in delivery systems encapsulating various anticancer drugs and natural plant products for targeting towards cervical cancer. The role of photothermal therapy, gene therapy and radiation therapy against cervical cancer is also discussed.

**Results:** Systemic/targeted drug delivery systems including liposomes, nanoparticles, hydrogels, dendrimers etc. and localized drug delivery systems like cervical patches, films, rings etc. are safer than the conventional chemotherapy which has further been proved by the several drug delivery systems undergoing clinical trials.

**Conclusion:** Novel approaches for the aggressive treatment of cervical cancer will optimistically result in decreased side effects as well as toxicity, frequency of administration of existing drugs, to overcome MDR and to increase the survival rates.

## 1. Introduction

Cervical cancer is the second most common cancer among women in the developing world, and the largest cancer killer among women in most developing countries, including India. Each year, over 500,000 women develop cervical cancer and about 275,000 women die from the disease [1]. By 2030, cervical cancer is expected to kill over 474,000 women per year, and over 95% of these deaths are expected to be in low- and middle-income countries [2]. The loss of these women – mothers, daughters, sisters, wives, partners, and friends – is almost entirely preventable. Although various drugs have been developed for cervical cancer, treatment with these drugs often results in a number of undesirable side effects, toxicity, and multidrug resistance (MDR). Therefore, novel approaches for the aggressive treatment of cervical cancer are urgently needed to decrease the side effects, toxicity, and frequency of administration of existing drugs, to overcome MDR, and to increase the survival rates [3].

The cervical cancer death rate is much lower in the USA and other developed countries. According to the National Cervical Cancer Coalitions, 80% of all cases of cervical cancer occur in developing countries. The American Cancer Society estimates that about 12,999 cases of invasive cervical cancer were diagnosed in the USA in 2016 and about 4,120 women died from the disease. The 5-year relative survival rate for the localized stage is 92% and the 5-year relative survival rate for all stages combined is 68% [4]. Recent disclosure by the World Health Organization (WHO) states that cervical cancer has emerged as the largest killer, surpassing breast cancer. Cervical cancer also emerged as a cancer having greater existence among the figures for both men and women put together. An estimated 73,000 new cases of cervical cancer have been reported in Indian women and the incidence reported is about 132,000 cases annually, i.e. about total 470,000 new cases of cervical cancer according to WHO. These deaths due to cervical cancer are largely preventable and likely to reduce significantly the cost burden of disease [5].

Cervical cancer is the term for a malignant neoplasm arising from cells originating in the cervix. In cervical cancer (cancer of the uterine cervix), cancer develops in the tissues of the cervix, which is part of the female reproductive system. The cervix connects the upper body of the uterus to the vagina. The endocervix (the upper part which is close to the uterus) is covered by glandular cells, and the ectocervix (the lower part which is close to the vagina) is covered by squamous cells. The transformation zone refers to the place where these two regions of the cervix meet. There are several types of cervical cancer, classified on the basis of where they develop in the cervix. Cancer that develops in the ectocervix is called squamous cell carcinoma, and around 80–90% of cervical cancer cases (more than 90% in India) are of this type [6,7]. Cancer that develops in the endocervix is called adenocarcinoma. Cervical cancer is caused by an invisible virus that is spread by having sex, just like human immunodeficiency virus (HIV). It is called human papillomavirus (HPV). Infection caused by HPV is the greatest risk factor for cervical cancer, followed by smoking [8]. Other risk factors include HIV. Although cancer of the cervix can develop in women of all ages, it usually develops in women aged 35–55 years, with the peak age for incidence varying with populations [9]. HPV infection and precancerous lesions go unnoticed and develop into full-blown cancer before women realize they need to go for medical help [10].

## 2. Pathophysiology

HPV is a DNA virus belonging to the newly named *papillomaviridae family* [11]. The papillomavirus induces proliferative lesions in the skin and internal mucosa. HPVs infect the genital mucosa that produce benign epithelial lesions and are the source of 90% of malignant carcinomas of the genital tract. Among 200 types of HPV, HPV 16 and 18 types are considered to be of ‘high risk’ and helpful in the progression of cervical cancer. HPV performs as a vector which confers susceptibility to neoplastic transmission or which incites direct transmutation to a malignant phenotype in some infected epithelial cells, and this transformation usually originates at the squamocolumnar junction of the cervix. *In situ* carcinoma is a condition in which all neoplastic cells of epithelial layers join the basement membrane. Progression of intraepithelial neoplasia to invasive disease usually takes 10–20 years. Most tumors (80–90%) exhibit squamous histology [12].

**Figure 1.**
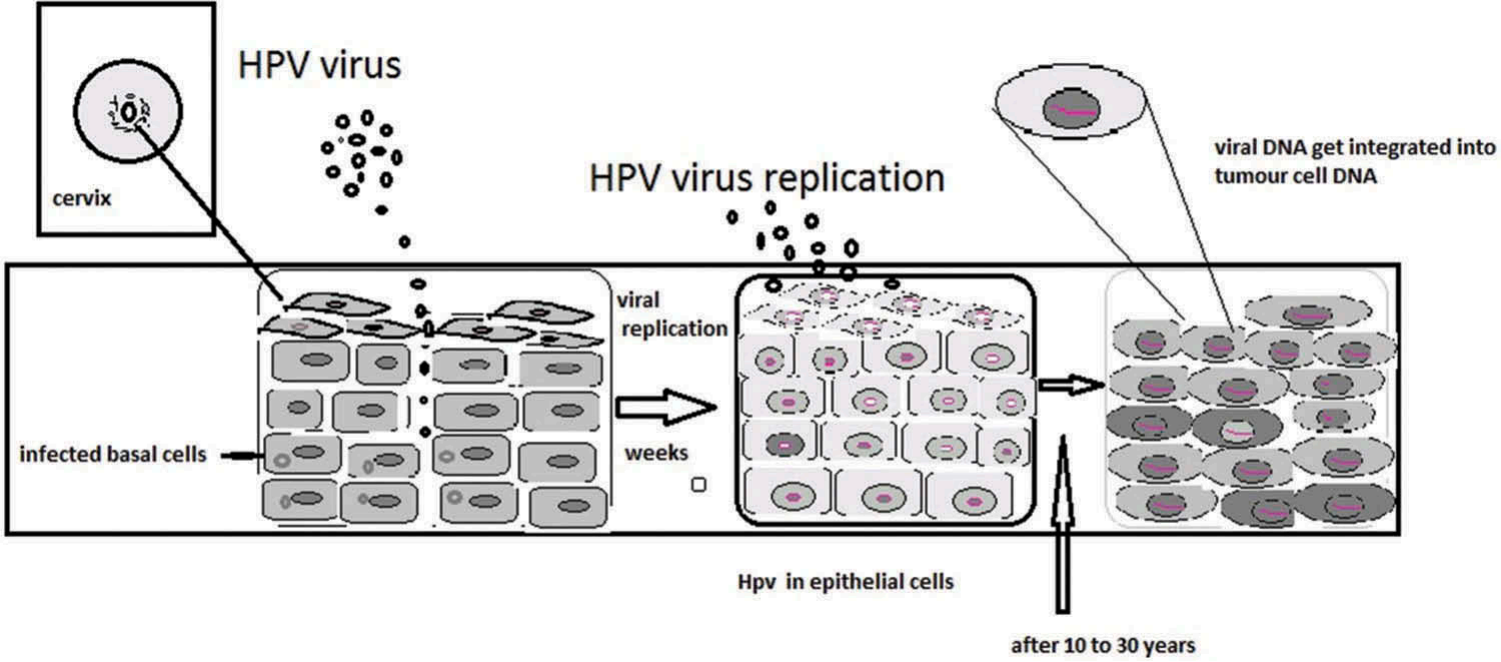
The mechanism of HPV infecting the host cells, its replication in epithelial cells, and integration into host cell’s DNA.

HPV primarily transmits by skin-to-skin contact via mild abrasion or micro-shock of the epidermis. It is assumed that the HPV replication cycle begins with entry of the virus inside basal cells of stratified squamous epithelium where HPV DNA replicates [13]. In the basal layer, viral replication is considered to be non-productive and the virus establishes itself as a low-copolymer episome by using the host DNA replication machinery to synthesize its DNA in differentiated keratinocytes. The virus switches to a rolling-circle mode of DNA replication that amplifies its DNA to high copy number synthesized capsid proteins and causes viral assembly. Infection of the cervical epithelium with oncogenic types of HPV and its precursor lesions (Figure 1) is vital in the development of cervical cancer. As per the literature epidemiologic profile, in 76% of cases women get cervical intraepithelial neoplasia (CIN) lesions attributed to sexually transmitted infection: more sexual partners, earlier age of first sexual intercourse, and lower socioeconomic status [14].

### 2.1. Transmission

**Figure 2.**
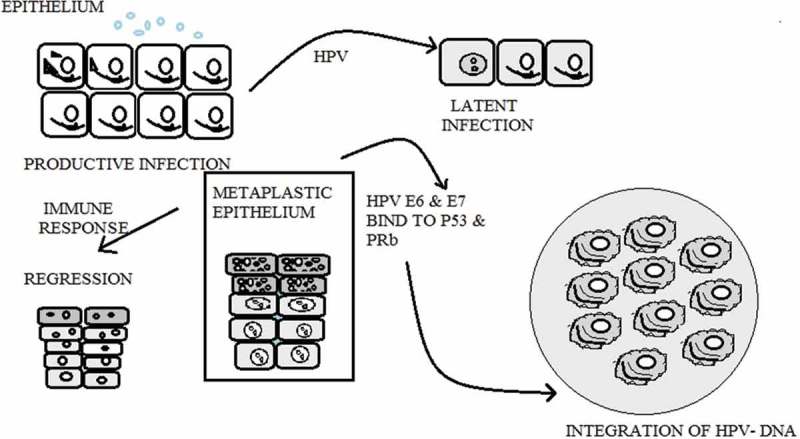
HPV virus productive phase, latent infection phase, regression phase, and integration of virus into host DNA.

The transmission of HPV occurs primarily by skin-to-skin contact. Basal cells of stratified squamous epithelium are first infected by HPV. Other cell types appear to be relatively resistant. The replication cycle of HPV begins with entry of the virus into the basal layer of the epithelium. Mild abrasion or microtrauma of the epidermis is required for HPV infection of the basal layer. After entering the host cell, viral DNA replicates. In the basal cells, replication of the virus is considered to be non-productive. The virus uses the host DNA replication machinery to synthesize its DNA on average once per cell cycle. In the keratinocytes of the suprabasal layer of the epithelium, the virus replicates with a high copy number of its DNA and capsid proteins are synthesized and cause viral assembly (Figure 2).

### 2.2. Mechanism

**Figure 3.**
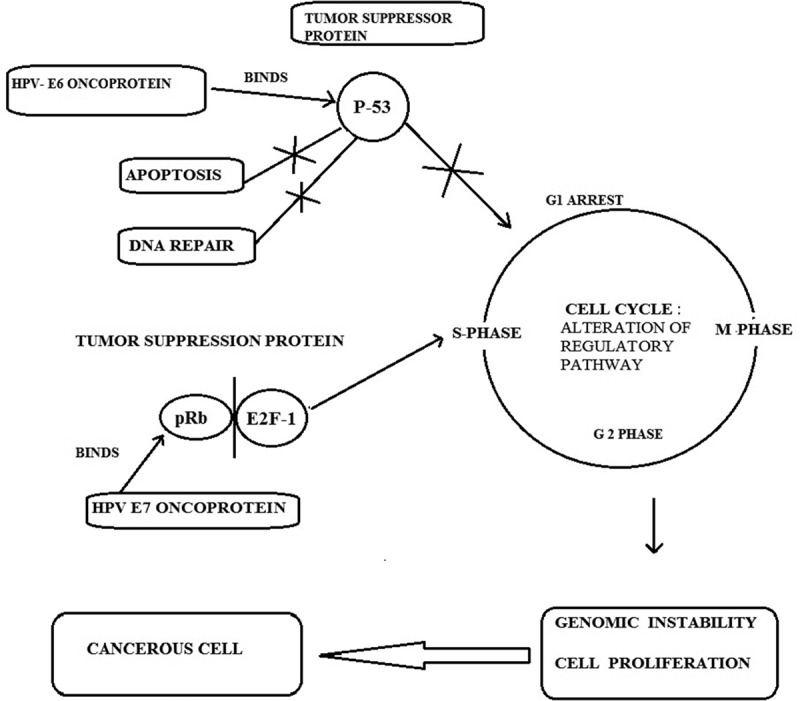
Schematic representation of the molecular mechanisms of oncogenic HPV infection, binding of E6 and E7 oncoprotien to the p53 and pRb genes, and blocking of apoptosis; G1 arrest which leads to genomic instability and neoplasia.

In the case of benign lesions caused by HPV, viral DNA is present extra-chromosomally in the nucleus. In invasive cancers, HPV-DNA is integrated into the host genome. Integration of viral DNA disrupts or deletes the E2 region, which causes the loss of its expression. This interferes with the function of E2, which normally down-regulates the transcription of the E6 and E7 genes and leads to an increased expression of E6 and E7 genes. The function of the E6 and E7 products during a productive HPV infection is to subvert the cell growth regulatory pathways and modify the cellular environment in order to facilitate viral replication. The E6 and E7 gene products deregulate the host cell growth cycle by binding and inactivating two tumor suppressor proteins: the tumor suppressor protein (p53) and the retinoblastoma gene product (pRb). The HPV E6 gene product binds to p53 and targets it for rapid degradation. As a consequence, the normal activities of p53, which govern G1 arrest, apoptosis, and DNA repair, are abrogated. Low-risk HPV E6 proteins do not bind p53 at detectable levels and have no effect on p53 stability *in vitro* (Figure 3) [15].

The HPV E7 gene product binds to pRb and this binding disrupts the complex between pRb and the cellular transcription factor E2F-1, resulting in the liberation of E2F-1, which allows the transcription of genes whose products are required for the cell to enter the S-phase of the cell cycle. The E7 gene product can also associate with other mitotically interactive cellular proteins such as cyclin E. The outcome is stimulation of cellular DNA synthesis and cell proliferation. The E7 protein from low-risk HPV types binds pRb with decreased affinity. Next, the E5 gene product induces an increase in mitogen-activated protein kinase activity, thereby enhancing cellular responses to growth and differentiation factors. This results in continuous proliferation and delayed differentiation of the host cell. The inactivation of p53 and pRb proteins can give rise to an increased proliferation rate and genomic instability. As a consequence, the host cell accumulates more and more damaged DNA that cannot be repaired, leading to transformed cancerous cells. In addition to the effects of activated oncogenes and chromosome instability, potential mechanisms contributing to transformation include methylation of viral and cellular DNA, telomerase activation, and hormonal and immunogenetic factors [16].

## 3. Chemotherapeutic drugs used in cervical cancer

Chemotherapy is the treatment of cancer with one or more cytotoxic antineoplastic drugs as part of a standardized regimen. Chemotherapy may be given with a curative intent or it may aim to prolong life or to palliate symptoms. It is often used in conjunction with other cancer treatments, such as radiation therapy or surgery. Systemic chemotherapy uses anticancer drugs that are given orally or injected intravenously. These drugs enter the bloodstream and reach all areas of the body. Conventional chemotherapy suffers some limitations.

1. Limited aqueous solubility: most chemotherapeutic agents are either from plant sources or are synthetic, which are hydrophobic and require solvents to formulate the dosages, which contributes to the severe toxicity.
2. Lack of selectivity of anticancer drugs: most chemotherapeutics lack selectivity toward cancerous cells, causing significant damage to rapidly proliferating normal cells.
3. Multidrug resistance: MDR was mainly due to increased efflux pumps such as P-glycoprotein (Pgp) in the cell membrane, which was responsible for transport of various anticancer drugs out of cells.

The drugs most often used to treat cervical cancer include cisplatin, paclitaxel, topotecan, ifosfamide, 5-fluorouracil (5-FU), docetaxel, mytomycin, epirubicin, and carboplatin (Table 1). Other drugs are used as well [17].

**Table 1.** Schematic representation of chemotherapeutic drugs used in cervical cancer with their class, mechanism of action, dose, and side effects.

Name of drugs	Structure	Class of drug	Mechanism of action	Dose	Side effects
Cisplatin (Cis-diaamine-di-chloroplatinum(II))		Antimetabolites	Platinum complexes react *in vivo*, bind to DNA and cause crosslinking of DNA, which ultimately triggers apoptosis. Of the bases on DNA, guanine is preferred. Subsequent to formation of [PtCl(guanine-DNA)(NH_3_)_2_]^+^, crosslinking can occur via displacement of the other chloride ligand, typically by another guanine	50–100 mg/m**^2^**	Nephrotoxicity, neurotoxicity, ototoxicity, electrolyte disturbance, hemolytic anemia
Carboplatin		Antimetabolites	Alkylating agents work: attachment of alkyl groups to DNA bases, resulting in the DNA being fragmented by repair enzymes in their attempts to replace the alkylating bases, preventing DNA synthesis and RNA transcription from the affected DNA	300 mg/m^2^	Hair loss, nausea, mild rash, mouth sores, constipation
Docetaxel		Taxol derivative	Docetaxel binds to the β-subunit of tubulin. Tubulin is the ‘building block’ of microtubules and the binding of docetaxel locks these building blocks in place	75 mg/m**^2^**	Severe diarrhea, yellowing of the skin or eye or stomach pain, swollen stomach, ankles or feet, numbness and tingling in the hands or feet (peripheral neuropathy)
Paclitaxel		Taxol	Paclitaxel enhances the polymerization of tubulin to stable microtubules and also interacts directly with microtubules, stabilizing them against depolymerization by cold and calcium, which readily depolymerize normal microtubules	135 mg/m**^2^** (in injection)	Neutropenia, hypersensitivity, hypotension, transient brady cardia, peripheral neuropathy, GI disturbances, alopecia
Epirubicin			Epirubicin forms complexes with DNA by intercalation between base pairs, and it inhibits topoisomerase II activity by stabilizing the DNA-topoisomerase II complex	Adult 60–90 mg/m**^2^**	Heart failure, swelling, ankles/feet, hair loss, nausea, allergic reaction
Fluorouracil		Antimetabolites	As a pyrimidine analogue, it is transformed inside the cell into different cytotoxic metabolites which are then incorporated into DNA and RNA, finally inducing cell cycle arrest and apoptosis by inhibiting the cell’s ability to synthesize DNA. It is an S-phase-specific drug and only active during certain cell cycles	3 mg/kg	Mouth sores, a sore throat, and trouble swallowing, diarrhea and stomach pain, decreased white blood cell counts, red blood cell counts and platelet counts can also be reduced, sun sensitivity and easy sunburning

## 4. Herbal drugs for the treatment of cervical cancer

Several drugs of plant origin have also been used extensively for cervical cancer chemotherapy. Hua-Nan Li et al. [18] investigated the antitumor effect of oroxylin A in the human cervical cancer HeLa cell line *in vitro* and *in vivo*. After being inoculated with the HeLa cells the mice were treated with oroxylin A and showed a significant decrease of tumor volumes and tumor weight compared with the control. Also, oroxylin A inhibited the growth of HeLa cells *in vitro* by MTT assay. The results showed that oroxylin A exhibited a strong antitumor effect both in mice and in the HeLa cell line, including apoptosis induction [19]. Moreover, Liu et al. [20] investigated and compared the anticancer effect of three new ganoderic acid derivatives on the cervical cell line HeLa. MTT assay indicated that the tested compounds, (22S,24E)-3α, 15α, 22-triacetoxy-5α-lanosta-7 and 9[11], 24-trien-26-oic acid amide (TLTO-A), displayed the highest inhibitory effect on the growth of HeLa cells and showed less cytotoxicity to the non-tumorous cell line MCF-10A than ganoderic acid. The apoptosis induction was presumed to occur through the endogenous pathway. The results showed both cytotoxic and pro-apoptotic effects of the compounds against HeLa cells.

In another study, Yao and Shulan [21] investigated the inhibitory effect of Guizhi-Fuling-decoction (GZFLD) on the invasive cervical cancer. Results showed that GZFLD inhibited the invasion of cervical cancer *in vitro* and *in vivo*. The inhibitory effects might be associated with restoring the matrix metalloproteinase tissue inhibitor (MMP–TIMP) balance, and then suppressing the degradation of extracellular matrix. Also, Fu et al. [22] studied sodium selenite, which can induce the apoptosis of cancer cells. MTT assay and morphological observation were used to study HeLa cells in order to obtain appropriate selenite concentrations for proteomic study. The results showed that selenite at concentration greater than 10 µmol/l significantly inhibited the viability of HeLa cells, and 40 µmol/l selenite was found to be appropriate for proteomic study. An increase in reactive oxygen species (ROS) generation and a decrease in mitochondrial membrane potential were detected in the selenite-treated cells compared with the control, which are consistent with the down-expression of antioxidative proteins in proteomics. This study also implies the potentiality of selenium in cervical cancer treatment.

Shen et al. [23] investigated proteomics in the human cervical cancer cell line HeLa treated with dicitratoytterbium (III) complex. Human cervical cancer cell line HeLa was found to be more sensitive to dicitratolanthanum (III) complex ([LaCit2]^3−^) than other cancer cell lines. Two-dimensional gel electrophoresis was one of the most powerful tools in proteomics studies. The biochemical and comparative proteomic analyses and [YbCit2]^3−^ were found to inhibit HeLa cell growth and induced apoptosis and were confirmed by the decreased mitochondrial transmembrane potential and the increased generation of ROS. These results suggested the mitochondrial pathway of cell apoptosis in [YbCit2]^3−^-treated cells, which will help in understanding the molecular mechanisms of lanthanide-induced apoptosis in tumor cells. Furthermore, Abdelwahab et al. [24] investigated the role of interleukin-6 (IL-6) and IL-6 receptors in the cytotoxic effects of zerumbone in ovarian and cervical cancer cell lines. Exposure of both cancer cells to zerumbone and cisplatin demonstrated the growth inhibition in a dose-dependent manner as determined by the MTT reduction assay. Zerumbone significantly decreased the levels of IL-6 secreted by both cancer cells. HeLa and Caov-3 cells were still sensitive to cisplatin and zerumbone in the presence of exogenous IL-6. The end result showed that the compound zerumbone inhibited cancer cell growth through the induction of apoptosis, arrested cell cycle at the G2/M phase, and inhibited the secretion levels of IL-6 in both cancer cells. Additionally, Kim et al. [25] investigated the antitumor activity of cytokine-induced killer (CIK) cells against human cervical carcinoma, generated CIK cells from human peripheral blood mononuclear cells (PBMC), and evaluated the antitumor activity of CIK cells both *in vitro* and *in vivo* in a nude mouse xenograft model. The results showed that an effector–target cell ratio of 100:1 destroyed 56% of CIK cells and KB-3-1 human cervical cancer cells. CD3^+^CD56^+^ cells from cultured human PBMC were expanded more than 1,000 times in the presence of anti-CD3 antibodies and IL-2 for 14 days. CIK cells broke down KB-3-1 cervical cancer cells *in vitro* and the adoptive transfer of CIK cells effectively prevented tumor growth in the nude mouse xenograft model. This study suggested that CIK cells were good candidates for cell immunotherapy in patients with cervical cancer. You et al. [26] studied gallic acid (GA), which is widely distributed in various plant and food products. They studied the effects of GA on HeLa cells in relation to cell growth inhibition and death. HeLa cell growth was inhibited with an IC_50_ of approximately 80 μM GA at 24 h whereas an IC_50_ of GA in human umbilical vein endothelial cells (HUVEC) was approximately 400 μM. Gallic acid-induced apoptosis and necrosis in HeLa cells and HUVEC were accompanied by the loss of mitochondrial membrane potential. The percentages of mitochondrial membrane potential loss cells and death cells were lower in HUVEC than in HeLa cells. It was concluded that GA inhibited the growth of HeLa cells and HUVEC via apoptosis and necrosis.

Ting et al. [27] studied arsenic trioxide (As_2_O_3_), which had therapeutic effects on cervical cancer by promoting apoptosis and inhibiting metastasis *in vitro* and *in vivo*. The growth inhibition properties and the combined effects of humic acid (HA) and As_2_O_3_ were studied in human cervical adenocarcinoma cell lines. The results showed that both As_2_O_3_ and HA inhibited the cell growth by ROS-mediated cell damage and activation of the apoptosis pathway. An enhanced antiproliferative action of As_2_O_3_ in HeLa and SiHa cells reduced the LC_50_ about 57.62 (300 μg HA/ml) to 83.67 (500 μg HA/ml), respectively. Additionally, Milrot et al. [28] investigated the mode of action of methyl jasmonate (MJ) in different cervical cancer cell lines. In addition to the short-term cytotoxicity, MJ reduced the survival of cervical cancer cells effectively by inducing apoptosis in all cervical cancer cells. MJ caused elevation of the mitochondrial superoxide anion in some cell lines, e.g. in HeLa and CaSki. Changes in the p53 and bax expression were variable, yet down-regulation of survivin was common with all cervical cancer cells. HPV E6 and E7 proteins were found to be reduced to significant levels by MJ without alteration of the mRNA levels. The cervical cancer cells having ectopic expression of E6, E7 that lack HPV (C33A) also showed a response to MJ. Thus, the study indicated MJ as an effective anticancer agent against different types of cervical cancer cell through altered pathways to induce cell death regardless of the presence of HPV.

Kniazhanski et al. [29] studied the effectiveness of MJ against cervical cancer cell lines. They showed that MJ is cytotoxic to a range of cervical cancer lines, including CaSki, SiHa, and HeLa, that carry HPV DNA and wild type C33A and p53 that is negative for HPV and contains mutant p53. The study concludes that MJ is a novel antitumor agent, acting via multiple pathways to induce death of cervical cancer cells, which makes it a promising candidate for treatment of cervical cancer.

## 5. Systemic versus localized drug delivery systems

**Figure 4.**
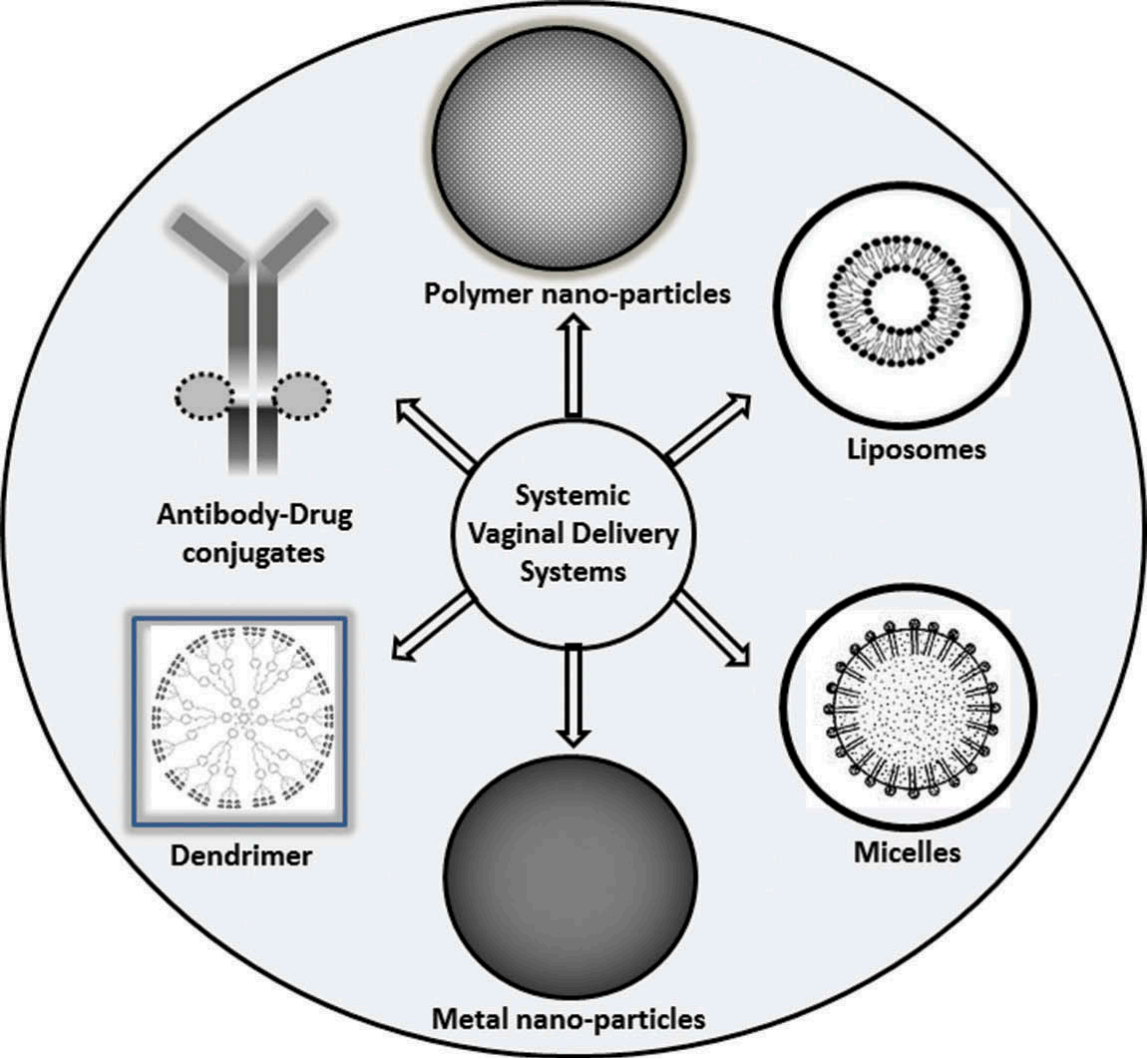
Various systemic drug delivery systems used in cervical cancer therapy.

Drug delivery approaches can be classified based on their route of administration (systemic or localized) and the type of device. Systemic delivery is based on particles/vesicles (dendrimers, micelles, liposomes, nano/microparticles) with surface features that assist in targeting the desired site when injected (Figure 4). The aim of a targeted drug delivery system is to localize, prolong, target, and have a protected drug interaction with the affected organ. The main advantages of the targeted drug delivery system are a reduction in frequency of the dosage taken, reduction of drug side effects, having a more uniform effect of the drug, and reduced fluctuation in blood concentration of drug. The targeted drug delivery system is highly integrated and its formation requires various disciplines, such as biology, chemistry, and engineering, to optimize this system [30]. Among the various approaches to targeting drug-loaded carrier complex to required sites in the body, the most advanced is passive (enhanced permeability and retention (EPR) effect-mediated) targeting, based on its accumulation in pathological sites with compromised vasculature. Nanoparticles of optimum size exhibit localization to specific organs or to diseased tissue via biological mechanisms, e.g. the RES (reticuloendothelial system) or the EPR effect are known as ‘passive targeting agents’. Specific targeting of the carriers could be achieved through the conjugation of carrier to a variety of ligands, including peptides, antibodies, aptamers, or other small molecules that possess high affinity for the disease sites such as cancer.

**Figure 5.**
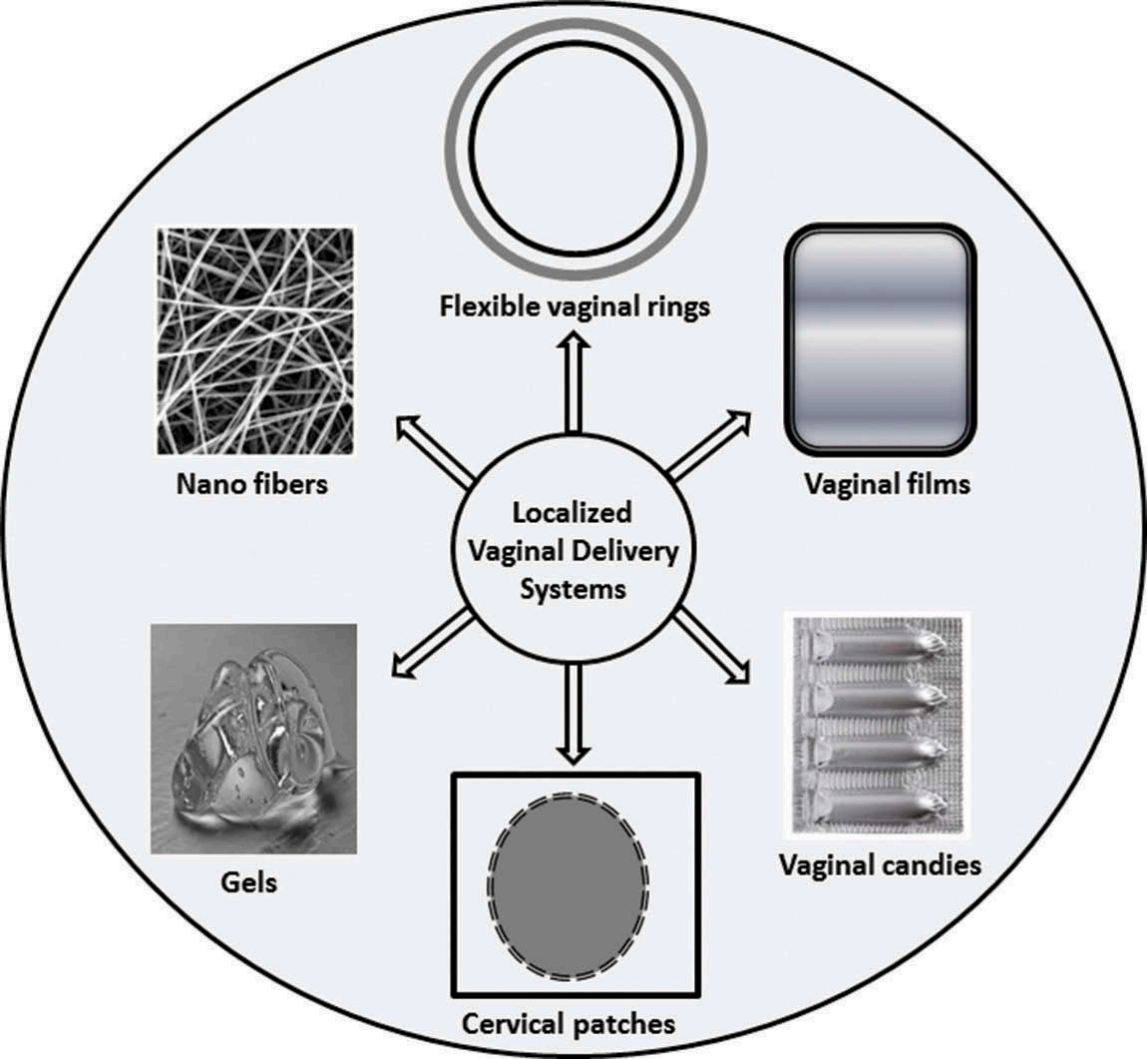
Various localized drug delivery systems used in cervical cancer therapy.

On the contrary, localized delivery limits systemic drug toxicities by direct delivery of the drug to the tumor (Figure 5). Based on depot systems that are implanted either directly into or adjacent to the tumor, the latter promotes the release of drug directly to the cancer site [31,32]. In recent years, numerous studies have been done with localized drug delivery strategies to treat cervical cancer. Although these strategies could reduce systemic toxicity, significant improvement in delivery strategies is still necessary to increase patient compliance and reduce chemotherapy-related side effects. Fortunately, the easily accessible cervix permits non-invasive implantation directly into the cancerous tissue at the time of brachytherapy implant [33].

The encapsulation of chemotherapeutic drugs in nanocarriers has gained substantial attention due to their structure, varied composition, and surface modifications [34]. The most common carriers for targeted drug delivery applications are nanoparticles, liposomes, micelles, and dendrimers. The size of these carriers ranges from 10 to 150 nm, which ensures increased accumulation in the tumor with longer circulation time. Particle sizes of less than 10 nm would be promptly cleared by the kidneys, and sizes larger than 150 nm would risk recognition and elimination by the macrophage cells [34]. There are a number of advantages of encapsulating chemotherapeutics in nanocarriers, such as protection from degradation in the bloodstream, enhancement of drug stability, targeted drug delivery, decreased toxic side effects, and improved bioavailability of the drug [35,36]. Nanoscale drug delivery systems have been revealed to augment drug specificity, reduce systemic drug toxicity, advance absorption rates, and offer shelter for active agents from biological and chemical degradation [37,38]. Additionally, they can be designed as controlled and/or sustained drug release systems to deliver therapeutics at a predetermined rate for a specified period of time. Controlled drug delivery systems could increase patient compliance by reducing the necessity for frequent drug administration [39]. Little is known about the effective concentrations of drugs used with the cervix tissue; however, it is recommended that controlled released formulations with longer release will increase the local concentration of the drugs and increase efficacy of the chemotherapies.

### 5.1. Liposomes

One of the most studied nanocarriers is liposomes [40]. Liposomes are one of the promising classes of nanomedicines, having the capability to exert site-specific chemotherapy, hence enhancing the quality of care for cancer patients. Nanomedicines implemented clinically for localized delivery are called first-generation liposomes. Stimuli-responsive liposomes having the ability to deliver site-specific chemotherapy as well as to provide triggered drug release are called second-generation liposomes and provide superior spatial and chronological run of therapy [41]. Stearyl amine-based positively charged multilayered liposomes loaded with paclitaxel (PTX) were prepared by a layer-by-layer technique. Prepared liposomes were coated with ‘anionic polyacrylic acid’ (PAA) and further by ‘cationic chitosan’. The chitosan-PAA-PTX liposomes exhibited noticeable sustained release performance, and enhanced PTX stimulated cytotoxicity in HeLa cell lines culture as compared with PTX liposomes [42].

Liposomal formulation containing cisplatin (lipoplatin) was developed to decrease the toxicity of cisplatin, to inhibit drug deposition at the tumor site, and to overcome the drug resistance. The study was conducted to analyze the antitumoral activity and mechanism of action of formulation in the ME-180 cervical cancer cell line and its cisplatin-resistant clone R-ME-180 and HeLa cells by means of cell proliferation assays, flow cytometry, ELISA assay, cell migration, spheroids, and tumor xenograft [43]. A novel heat-activated thermosensitive liposome formulation containing cisplatin was developed to release around 90% of the loaded drug within 5 min in mild heating conditions. The results indicated that the heat-activated, triggered release formulation of cisplatin can improve the therapeutic index of the drug [44]. Also, doxorubicin liposomes (Lipo-DOX) were developed and studied at a dose of 20 mg/m^2^ in formerly treated patients with recurrent cervical cancer as a Simon’s two-stage Phase II clinical trial. The study included 10 patients with recurrent cervical cancer. Lipo-DOX with a predecided intravenous dose was diluted in 250 ml of 5% dextrose solution and administered at intervals of 2 weeks. Salvage chemotherapy with Lipo-DOX showed partial response in patients with recurrent cervical cancer [45].

Corona-Ortega et al. [46] developed anionic, cationic and neutral liposomes containing IL-2 to assess their affinity to a cervical cancer cell line (INBL) and also to decide whether they can present IL-2 on their external surface. Anionic liposomes were found to be cytotoxic upon co-culturing with cervical cancer cell line, and neutral, cationic liposomes were identified by using a fluorescent anti-IL-2 antibody. Strong IL-2 was found on the cell membranes, demonstrating higher affinity of the liposomes to the INBL cells. Moreover, doxorubicin-loaded stealth liposomes (Tf-SL-DOX) were prepared by means of a film dispersion method and, further, by the ammonium sulfate gradient method. Later, transferrin (Tf) was conjugated to the liposome surface by an amide bond between DSPE-PEG2000-COOH and Tf. It was concluded that stealth liposomes are efficient carriers for drugs, genes, and vaccines and can be easily tailored with proteins, antibodies, and other suitable ligands, resulting in smart formulations for targeted drug delivery [47]. Furthermore, liposomes conjugated with folic acid and transferrin showed higher cell association, penetration, and efficacy of delivering doxorubicin *in vitro* in human cervical carcinoma cell line (HeLa) as compared with either of the single-ligand targeted liposomes, or non-targeted liposomes [48].

Nonetheless, Corona et al. [49] prepared cationic liposomes containing IL-2 (CL-IL-2) which form a stable complex by interacting with the negatively charged DNA molecules and cell membranes. The dose, 300 μl of either CL-IL-2 or empty liposomes in PBS or only PBS, was administered intraperitoneally daily for 5 days in INBL cells induced tumor-containing immune-depressed CBA mice, and the tumor masses were evaluated. The study showed that PBS did not show any effect on the tumors, whereas empty liposomes showed a total tumor reduction of 50% and CL-IL-2 showed a total tumor reduction of 94%.

The aforementioned liposomal delivery systems are summarized in Table 2.

**Table 2.** Liposome-based delivery systems, including cell lines/animal models used for cervical cancer therapy.

S. No.	Drug/antibody	Type/composition of liposomes	Cell lines/animal/clinical models	Reference
1.	Paclitaxel	Stearyl amine-based positively charged multilayered liposomes	HeLa cell lines	[42]
2.	Cisplatin	–	ME-180 cervical cancer cell line, cisplatin-resistant clone R-ME-180 and HeLa cells	[43]
3.	Cisplatin	Heat-activated thermosensitive liposome	–	[44]
4.	Doxorubicin	–	Cervical cancer patients	[45]
5.	IL-2	Anionic, cationic, and neutral liposomes	INBL cells	
6.	Doxorubicin	Transferrin (Tf)-conjugated stealth liposomes (Tf-SL-DOX)	–	[47]
7.	Doxorubicin	Liposomes conjugated with folic acid and transferrin	HeLa cells	[48]
8.	IL-2	Cationic liposome containing	INBL cells induced tumor-containing immune-depressed CBA mice	[49]

### 5.2. Nanoparticles

Nanoparticles can be demarcated as ultra-dispersed solid supramolecular structures with size ranging between 10 and 1000 nm. Drugs can be encapsulated, entrapped, dissolved, or attached to a nanoparticle matrix which acts as a reservoir, and nanoparticles have a prospective influence in the treatment of cancer. The conjugated gold nanoparticles encapsulating hydrophobic protoporphyrin were found to be a potential carrier for photodynamic therapy against cervical cancer. Docetaxel-loaded PCL-PLA-TPGS copolymer ‘[poly(**ɛ**-caprolactoneco-lactide)-D-α-tocopheryl polyethylene glycol succinate]’ nanoparticles showed more effectiveness in the reduction of cervical cancer cell number in comparison with taxotere after 48 and 72 h treatment during *in vitro* cancer cell viability experiments [50]. Nanoparticles prepared using a new molecular biomaterial, a star-shaped block copolymer, i.e. colic acid-PLGA-b-Vitamin E TPGS copolymer, for controlled delivery of docetaxel were found to possess highest antitumor efficacy and cellular uptake efficiency when compared with poly(lactic-co-glycolic) acid (PLGA) nanoparticles and PLGA-b-TPGS nanoparticles [51]. Cisplatin, when entrapped in non-aggregated folic acid-conjugated gelatin nanoparticles to improve drug delivery for the treatment of cancer, has shown a higher cellular uptake of 81% when compared with cisplatin plain gelatin nanoparticles, which have 51% uptake [52]. Vivero-Escoto et al. [53] synthesized phenanthridinium (oligonucleotide intercalator) functionalized mesoporous silica nanoparticles and found that the prepared nanoparticles have austere cell growth inhibition due to the binding of the phenanthridium group present on the peripheral surface to cytoplasmic oligonucleotides such as HeLa cell messenger RNAs. They also anticipated that, having good biocompatibility and trafficking of cell membrane properties, the nanoparticles can be further used for numerous biomedical applications. Bleomycin sulfate-loaded nanostructured lipid particles resulted in improved oral bioavailability by avoiding first-pass metabolism and thereby upsurges in intestinal lymphatic uptake of drug to reach systemic circulation, finally leading to greater toxicity and apoptosis against cervical cancer cells [54]. Doxorubicin-encapsulated long circulating self-assembled nanoparticles prepared using amphiphilic brush-like block copolymer composed of polynorbonene-cholesterol/poly(ethylene glycol) were found to display considerably greater inhibition of tumor growth as compared with free doxorubicin and it was concluded that the prepared nanoparticles would be a valuable carrier for improving tumor drug delivery [55]. Confocal laser scanning microscopy (CLSM) revealed that doxorubicin hydrochloride-loaded pH-responsive charge reversal, polymer-coated mesoporous silica nanoparticles resulted in effective delivery and release of doxorubicin hydrocholride to the nucleus of HeLa cells [56]. Docetaxel-encapsulated ‘D-α-tocopheryl polyethylene glycol 1000 succinate-b-poly(ε-caprolactone-ran-lactide)’ nanoparticles prepared using a modified nanoprecipitation method were found to demonstrate higher cytotoxicity and tumor growth inhibition on a xenograft BALB/c nude mice tumor model as compared with taxotere and therefore it was concluded that this polymer can be a novel polymeric material for nanoformulation [57]. Cyclodextrin-containing pH-sensitive poly(2-(dimethylamino) ethyl methacrylate) star polymer nanoparticles encapsulating doxorubicin were prepared, which resulted in higher cytotoxicity and cellular uptake and could effectively suppress the tumor growth without significant side effects [58]. Transferrin-conjugated amphipillic poly(γ-glutamic acid-maleimide-co-l-lactide)-1,2-dipalmitoylsn-glycero-3-phosphoethanolamine (γ-PGA-MAL-PLADPPE) copolymer-based targeted nanoparticles loaded with PTX resulted in increased PTX activity due to the presence of transferrin and could also assist tumor-specific therapy [59].

Docetaxel-loaded biocompatible amphiphilic pentablock copolymeric nanoparticles synthesized using poly(lactide-co-glycolide) and pluronic F68 by emulsion solvent evaporation and simple dialysis showed a significant cytotoxicity against cervical cancer cells [60]. A combination therapy consisting of multidrug-incorporated layered double hydroxide nanohybrids has been proved to be an efficient treatment for cervical cancer. Kim et al. [61] developed dual drug (methotrexate and 5-flurouracil)-loaded layered double hydroxide nanoparticles which showed increased anticancer efficacy of dual drug nanohybrids when compared with free drugs and single drug nanohybrids. Bioadhesive pellets of hexylaminolevulinate (HAL) containing Carbopol 934 were developed by Hiorth et al. [62] using a spheronization technique for photodynamic therapy of cervical cancer, and the prepared pellets were found to be mechanically stable with good bioadhesivity and proficient HAL release, thus proving to be an efficient therapy for cervical cancer. Moreover, Sarisozen et al. [63] fabricated transferrin-modified polyethylene glycol phosphatidyl ethanolamine (PEG-PE)-based polymeric micelles for co-delivery of PTX and curcumin, which were assessed for cytotoxicity, cellular accumulation, and association against spheroids of multidrug-resistant ovarian cancer cells and *in vivo* tumor models using confocal imaging and flow cytometry. The results indicated that micelles penetrate significantly deeper in spheroids, delivering cytotoxic agents efficiently and thereby enhancing the cytotoxicity. The *in vivo* results demonstrate the applicability of the system for cervical cancer therapy.

Tan and coworkers [64,65] prepared doxorubicin-loaded folate (FA)-modified carboxymethyl chitosan (FCC) hydrogel nanoparticles which were used as a FA-receptor-targeted drug carrier for tumor-specific drug delivery. Cellular uptake of FCC nanoparticles was found to be higher than that of nanoparticles based on linoleic acid (LA)-modified carboxymethyl chitosan because of the FA-receptor-mediated cellular uptake, thereby providing higher cytotoxicity against HeLa cells. Additionally, Danhier et al. [66] developed Cremophor EL-free nanoparticles loaded with PTX by simple emulsion and nanoprecipitation. The antitumoral activity was assessed using the HeLa cell line by the MTT assay and was compared with the commercial formulation Taxol and with Cremophor EL. When exposed to PTX (25 µg/ml), the cell viability was lower for PTX-loaded nanoparticles than for Taxol (IC_50_ 5.5 vs. 15.5 µg/ml). HeLa cells when exposed to Taxol and PTX-loaded nanoparticles induced the same percentage of apoptotic cells. A greater *in vivo* tumor growth inhibition effect was observed with PTX-loaded nanoparticles as compared with Taxol. Thus, the PTX-loaded nanoparticles might be considered as an effective anticancer drug delivery system for cancer chemotherapy.

Yuandong et al. [67] synthesized a novel biodegradable docetaxel-loaded PLGA-TPGS, a random copolymer from lactide, glycolide and D-α-tocopheryl polyethylene glycol 1000 succinate (TPGS) nanoparticles. *In vitro* cellular uptakes of such nanoparticles were investigated in human cervix carcinoma cells (HeLa) by using CLSM, demonstrating fluorescence. The developed nanoparticles had significant cytotoxicity against HeLa cells, which was in a time- and concentration-dependent manner. The study concludes that PLGA-TPGS random copolymer-based nanoparticles are efficient drug delivery systems for cancer chemotherapy. In another study, Kim et al. [68] developed PTX-loaded PEG/PCL biotin-conjugated nanoparticles which had no significant adverse effects on cell viability at 0.005–1.0 µg/ml regardless of cell type (normal human fibroblasts and HeLa cells). Also, conjugated nanoparticles showed a much higher cytotoxicity for cancer cells than was observed in the PEG/PCL nanoparticles without the biotin group. These results reveal that the biotin-conjugated nanoparticles could improve the selective delivery of PTX into cancer cells via interactions with overexpressed biotin receptors on the surfaces of cancer cells. Gu et al. [69] prepared PTX-loaded nanoparticles, and a cytotoxicity assay was performed using breast cancer MCF-7 and cervical cancer HeLa cells. The half maximal inhibitory concentrations (IC_50_) of PTX nanoparticles and PTX were found to be 8.5 ± 0.3 and 14.0 ± 0.7 ng/ml at 48 h and 3.5 ± 0.4 and 5.2 ± 0.5 ng/ml at 72 h across several runs. The IC_50_ of PTX nanoparticles and PTX for HeLa cells were found to be 5.0 ± 0.3 and 8.0 ± 0.3 ng/ml at 48 h and 2.0 ± 0.1 and 6.5 ± 0.3 ng/ml at 72 h. Thus, PTX-loaded nanoparticles were found to be an efficient carrier for site-specific delivery.

Zhang et al. [70] prepared doxorubicin-loaded self-assembled oleoyl-chitosan (OCH) nanoparticles. The *in vitro* toxicity profile of OCH nanoparticles was evaluated by hemolysis test and MTT assay. The OCH nanoparticles showed no cytotoxicity to mouse embryo fibroblasts. The drug was rapidly and completely released from the nanoparticles at pH 3.8, whereas sustained release was obtained at pH 7.4. The inhibition rate was 48.67% with a concentration of 10 µg/ml after 1 day and 68.72% for the second day on HeLa cells. These results reveal the potential of OCH nanoparticles as carriers for hydrophobic antitumor agents. Furthermore, docetaxel-loaded biodegradable D-α-tocopheryl polyethylene glycol 1000 succinate-b-poly(ε-caprolactone-ran-glycolide) (TPGS-b-(PCL-ran-PGA)) nanoparticles were prepared and further modified by polyethyleneimine for coating plasmid pShuttle2-endostatin for the synergistic treatment of cervical cancer. The results of fluorescent microscopy, CLSM, western blot analysis, and MTT assay showed that the TPGS-b-(PCL-ran-PGA)/PEI nanoparticles can efficiently and simultaneously deliver both Coumarin-6 and plasmids into HeLa cells, and the expression of endostatin was verified via western blot analysis. Compared with control groups, the TPGS-b-(PCL-ran-PGA)/PEI-pShuttle2-endostatin nanoparticles significantly decreased the cell viability of HeLa cells (*p* < 0.01), inhibited the growth of tumors, and even eradicated the tumors. The underlying mechanism is attributed to synergistic antitumor effects by the combined use of docetaxel, endostatin, and TPGS released from nanoparticles. The TPGS-b-(PCL-ran-PGA) nanoparticles could function as multifunctional carriers for chemotherapeutic drugs and genetic material delivery, and offer substantial potential as a model candidate for *in vivo* cancer therapy [71].

Wang et al. [72] prepared the docetaxel-loaded D-α-tocopheryl polyethylene glycol 1000 succinate-b-poly(ε-caprolactone-ran-lactide) (TPGS-b-(PCL-ran-PLA)) nanoparticles and evaluated their therapeutic effects in comparison with taxotere both *in vitro* and *in vivo*. TPGS-b-(PCL-ran-PLA) nanoparticles showed an obvious increase of cellular uptake. Owing to the advantages of TPGS-b-(PCL-ran-PLA) nanoparticles, it could achieve a significantly higher level of cytotoxicity *in vitro* and better inhibition effect of tumor growth on a xenograft BALB/c nude mice tumor model than commercial taxotere at the same dose (1.49-fold more effective). The TPGS-b-(PCL-ran-PLA) could be used as a novel and potentially biodegradable polymeric material for nanoformulation in cervical cancer chemotherapy. Pimentel et al. [73] developed silver nanoparticles (AgNPs) and further encapsulated them in nanocarriers (AgNPs-NT) with a biocompatible surface based on polyethylene glycol for cancer chemotherapy. Different doses of free and encapsulated AgNPs were evaluated *in vitro* on the cervical cancer-derived cell lines HeLa and CaSki, by flow cytometry studies at 24 h, employing propidium iodide (PI) and carboxyfluorescein diacetate succinimidyl ester (CFSE) as reporter molecules. Both free and encapsulated AgNPs were found to be toxic for both cell lines, inducing important decrements on the cell viability compared with cisplatin (positive control, 0.250 mM) and the negative control (NCtrl) comprising only the cell culture media, the vehicle of the AgNPs (0.360 mM), and the vehicle of the AgNPs-NT (1.089 mM). In both cases (HeLa and CaSki cells), AgNPs-NT seems to be less effective than free AgNPs, but this AgNPs-NT had a biocompatible surface, adequate size, and high toxicity observed *in vitro*.

Folate receptor is a potential therapeutic target in cervical cancer overexpressed in human cervical cancer cells [74]. Nanoparticles that were conjugated with folic acid to L-tyrosine-polyphosphate [75], chitosan [76], or chitosan-coated PLGA nanoparticles [77] and loaded with silver carbene complex, cisplatin, selenocystine, or carboplatin, respectively, increased the specificity of chemotherapeutic drugs up to 10-fold more than control nanoparticles without drug in cervical cancer cells. Folate-targeted doxorubicin-loaded nanoparticles exhibited improved targeting and antitumor efficacy in inhibiting tumor cells *in vivo* [74]. In a recent study, pullulan acetate nanoparticles decorated with folate were used as a carrier for treating cervical carcinoma and its metastatic hepatocellular carcinoma [78].

The aforementioned nanoparticle-based drug delivery systems are summarized in Table 3.

**Table 3.** Nanoparticle-based delivery systems, including cell lines/animal models used for cervical cancer therapy.

S. No.	Drug	Type/composition of nanoparticles	Cell lines/animal models/neoplasm	References
1.	Docetaxel	Poly(**ɛ**-caprolactoneco-lactide)-D-α-tocopheryl polyethylene glycol succinate nanoparticles	Cervical cancer cells	[50]
2.	Docetaxel	Colic acid-PLGA-b-Vitamin E TPGS copolymer	–	[51]
3.	Cisplatin	Folic acid-conjugated gelatin nanoparticles	–	[52]
4.	–	Phenanthridinium (oligonucleotide intercalator)-functionalized mesoporous silica nanoparticles	HeLa cells	[53]
5.	Bleomycin sulfate	Nanostructured lipid particles	Cervical cancer cells	[54]
6.	Doxorubicin	Polynorbonene-cholesterol/poly(ethylene glycol)	–	[55]
7.	Doxorubicin	Mesoporous silica nanoparticles	HeLa cells	[56]
8.	Docetaxel	D-α-tocopheryl polyethylene glycol 1000 succinate-b-poly(ε-caprolactone-ran-lactide) nanoparticles	Xenograft BALB/c nude mice tumor model	[57]
9.	Doxorubicin	Cyclodextrin-containing pH-sensitive poly(2-(dimethylamino) ethyl methacrylate) star polymer nanoparticles	–	[58]
10.	Paclitaxel	Poly(γ-glutamic acid-maleimide-co-l-lactide)-1,2-dipalmitoylsn-glycero-3-phosphoethanolamine (γ-PGA-MAL-PLADPPE) copolymer-based nanoparticles	–	[59]
11.	Docetaxel	Poly(lactide-co-glycolide) and pluronic F68	Cervical cancer cells	[60]
12.	Methotrexate and 5-flurouracil	Double hydroxide nanoparticles	–	[61]
13.	Hexylaminolevulinate (HAL)	Bioadhesive pellets of Carbopol 934	Cervical cancer cells	[62]
14.	Paclitaxel and curcumin	Polyethylene gycol phosphatidyl ethanolamine (PEG-PE)-based polymeric micelles	Multidrug-resistant ovarian cancer cells	[63]
15.	Doxorubicin	Folate (FA)-modified carboxymethyl chitosan (FCC)	HeLa cells	[64,65]
16.	Paclitaxel	Cremophor EL-free nanoparticles	HeLa cells	[66]
17.	Docetaxel	PLGA-TPGS, glycolide and D-α-tocopheryl polyethylene glycol 1000 succinate (TPGS) nanoparticles	HeLa cells	[67]
18.	Paclitaxel	PEG/PCL biotin-conjugated nanoparticles	Normal human fibroblasts and HeLa cells	[68]
19.	Paclitaxel	Paclitaxel-loaded nanoparticles	MCF-7 and HeLa cells	[69]
20.	Doxorubicin	Oleoyl-chitosan (OCH) nanoparticles	HeLa cells	[70]
21.	Docetaxel	D-α-tocopheryl polyethylene glycol 1000 succinate-b-poly(ε-caprolactone-ran-glycolide) nanoparticles	HeLa cells	[71]
22.	Docetaxel	D-α-tocopheryl polyethylene glycol 1000 succinate-b-poly(ε-caprolactone-ran-lactide) nanoparticles	Xenograft BALB/c nude mice tumor model	[72]
23.	Silver nanoparticles	Polyethylene glycol	HeLa and CaSki cells	[73]

### 5.3. Nanotubes

Nanotubes belong to the fullerene structural family. Their name is derived from their hollow, long structure with one-atom-thick sheets like walls formed of carbon, called graphene. The properties of nanotubes depend on specific and discrete (‘chiral’) angles, rolling angle, and radius. Nanotubes are categorized on the basis of number of walls, e.g. as single-walled nanotubes (SWNTs) and multi-walled nanotubes (MWNTs) [79]. Individual nanotubes align themselves naturally into rope-like structures held together by van der Waals forces. Applied quantum chemistry (orbital hybridization) best describes chemical bonding taking place in nanotubes. The nanotube is composed entirely of sp2 hybridized bonds, as found in graphite. These bonds provide nanotubes with their unique strength. Owing to their flexibility and strength, the carbon nanotubes have an important role in nanotechnology engineering [80,81].

Zhang et al. [82] developed a pH-based targeted drug delivery system composed of single-walled carbon nanotubes (SWCNTs), derivatized with carboxylate groups, and coated with a polysaccharide material, loaded with the anticancer drug doxorubicin (DOX), which was bound at pH 7.4 and released at a lower pH, lysosomal pH, and pH environment of certain tumors. Folic acid, a targeting agent, was anchored to the SWCNTs to selectively deliver DOX into the lysosomes of HeLa cells with much higher efficiency than free DOX. The drug (dox) released from the modified nanotubes damaged nuclear DNA and inhibited cell proliferation.

Mahmood et al. [83] investigated the use of carbon nanotubes (CNTs) in combination with conventional drugs, which enhanced their chemotherapeutic effects. HeLa and human Panc1 cancer cells were treated with CNTs (24 h, 10 and 20 µg/ml), etoposide (6 h, 75 × 10^−^^6^ M), and their combination. The various methods, e.g. flow cytometry, caspase-3 assay and Trypan blue dye, were used to control cell viability. Antitumor activity of the combination of etoposide and CNTs against cancer cells was found to be much higher as compared with the administration of etoposide and CNTs alone. Data provided by viability assays suggest that there is a strong interaction between CNTs and the cellular structures which improved the effectiveness of conventional chemotherapeutic agents by synergistic curative rates. Moreover, Bhatnagar et al. [84] prepared functionalized SWCNTs conjugated with chitooligosaccharide (f-SWNT-COS) as a drug delivery system. In addition, drug gliotoxin (GTX) and targeting molecules (lysozyme, p53, and folic acid) were incorporated into f-SWNT-COS. f-SWNTs-COS-GTX-p53, f-SWNTs-COS-GTX-lysozyme, and f-SWNTs-COS-GTX-FA showed significant cytotoxicity against cervical cancer (HeLa) cells and breast cancer (MCF-7) cells, and f-SWNTs-COS-GTX-p53 was found to be the most effective delivery vehicle with a controlled release and enhanced cytotoxicity rendered through apoptosis in HeLa cells.

### 5.4. Micelles

Polymeric micelles are colloidal particles made up of amphiphilic block copolymers that can assemble themselves [85]. They are imperative for cancer therapeutic applications due to their *in vivo* stability, ability to solubilize water-insoluble drugs, prolongation of blood circulation time, and small size of 10–100 nm [86]. Folic acid-conjugated, PTX-loaded polymeric composite micelles targeted and inhibited tumor growth and caused cell apoptosis of U14 cervical cancer tumors both *in vitro* and *in vivo* [87]. Also, PTX-loaded polymeric micelles of candesartan-g-polyethyleneimine-cis-1,2-cyclohexanedicarboxylic anhydride polymer with negative surface charges revealed strong antitumor efficacy by facilitating amidase-responsive drug-release manners and rapid endosomal escape [88].

De Melo-Diogo et al. [89] fabricated ‘D-α-tocopheryl polyethylene glycol 1000 succinate-poly(lactic acid)’ (TPGS-PLA)-based micelles encapsulating sildenafil (intracellular drug accumulation enhancer) along with two anticancer agents (Palbociclib and Crizotinib) for enhanced cytotoxic effect. Triple drug-loaded TPGS-PLA micelles showed enhanced cytotoxicity in comparison with single or double drug combinative micelles against A549 lung cancer cells and reported as novel drug carriers for multiple drug therapy in cancer treatment. Feng et al. [90] developed two different kinds of PTX-conjugated micelle, in which one contained 25% (mass fraction) PTX [M (PTX)] and the other contained 22.5% (mass fraction) of PTX and 1.4% (mass fraction) of folate (FA) [FA-M (PTX)], and evaluated them for cell apoptosis and antitumor activity using U14 cervical cancer mouse models in comparison with 0.9% (mass fraction) saline (control) and equivalent Taxol. The mice were killed to measure the tumor masses, 7 days post-tail intravenous injection of the drugs. The average tumor masses for different formulations, e.g. control, Taxol, M (PTX) and FA-M (PTX), were measured to be 4.26, 2.89, 2.63, and 2.17g, respectively, with 32, 38, and 49% rates of inhibition of tumor growth for the three drug groups, respectively. The rate of cell apoptosis based on the flow cytometry data and the terminal deoxynucleotidyl transferase (TdT)-mediated deoxyuridine triphosphate (dUTP) nick end labeling (TUNEL) assay data were 20, 31, 37, 42%, and 10, 22, 26, 34%, respectively. This showed that the FA-M (PTX) had maximum antitumor activity against HeLa cells.

### 5.5. Monoclonal antibodies

Monoclonal antibodies (mAb or moAb) are monospecific antibodies that are the same because they are obtained from identical immune cells that are all clones of a unique parent cell, as compared with polyclonal antibodies which are made from several different immune cells. Monoclonal antibody therapy includes the use of mAb to bind specifically to target cells or proteins, which then stimulate the patient’s immune system to attack those specific cells. It is possible to create a mAb specific for almost any extracellular/cell surface target, thus a huge amount of research and development is currently being undertaken to create monoclonals for various serious diseases (such as multiple sclerosis, rheumatoid arthritis, and different types of cancer). For example: it can be used to destroy malignant tumor cells and check tumor growth by blocking specific cell receptors; and in radioimmunotherapy, where a radioactive dose localizes on a target cell line, delivering chemical doses to the target [91]. The advent of this technology has made it possible to raise antibodies against specific antigens presented on the surfaces of tumors [92].

Meira et al. [93] investigated anti-EGFR monoclonal antibodies including cetuximab’s toxicity plus chemoradiation on cervical cancer cells, expressing different EGFR (epidermal growth factor receptor) levels using western blotting and MTT or clonogenic assays. The results showed that cetuximab with cisplatin and radiation achieved maximum cytotoxic effects for CaSki, A431, and C33A cells. Cetuximab efficiently decreased MAPK and AKT phosphorylation in A431 cells, but slightly less in CaSki and C33A cells. To check whether further EGFR, MAPK, or HER2 inhibition would improve cetuximab’s cytotoxicity, the researchers combined it with an EGFR tyrosine kinase inhibitor (TKI), trastuzumab, or a MEK1/2 inhibitor (PD98059). In CaSki, but not in C33A cells, cetuximab combined with the TKI, reducing cell survival and MAPK and AKT phosphorylation. The cetuximab and trastuzumab or PD98059 reduced survival and MAPK phosphorylation of both cell lines. Data indicated that cetuximab combined with chemoradiation, trastuzumab, or MAPK inhibitors is useful for cervical cancer treatment, independently of EGFR expression. Furthermore, Monk et al. [94] studied pazopanib and lapatinib (tyrosine kinase inhibitors), which target platelet-derived growth factor receptor, vascular endothelial growth factor receptor (vEGFR) and EGFR and human epidermal growth factor receptor 2 (HER2/neu), respectively. In cervical cancer, EGFR, HER2, and high microvascular density correlate with survival. Patients with measurable stage IVB persistent/recurrent cervical carcinoma not responsive to curative therapy and at least one prior regimen in the metastatic setting were randomly assigned in a ratio of 1:1:1 to pazopanib at 800 mg once daily, lapatinib at 1,500 mg once daily, or pazopanib plus lapatinib combination therapy (pazopanib at 400 mg plus lapatinib at 1,000 mg once daily or lapatinib at 1,500 mg plus pazopanib at 800 mg once daily). This study confirmed the activity of antiangiogenesis agents in advanced and recurrent cervical cancer and demonstrated the benefit of pazopanib based on the prolonged progression-free survival and favourable toxicity profile.

Shen et al. [95] investigated lanthanides, which have been reported to induce apoptosis in cancer cell lines. HeLa was found to be more sensitive to dicitratolanthanum (III) complex than other cancer cell lines. By using biochemical and comparative (proteomic) analyses, [YbCit2]^3−^ was found to inhibit growth of HeLa cells and induce apoptosis. Proteomics results from [YbCit2]^3−^-treated cells revealed profound changes in proteins of mitochondria and oxidative stress, as with [LaCit2]^3–^, suggesting that mitochondrial dysfunction played a key role in [YbCit2]^3−^-induced apoptosis. The results suggested a mitochondrial pathway of cell apoptosis in [YbCit2]^3−^-treated cells, which expressed molecular mechanisms of lanthanide-induced apoptosis in tumor cells. In another study, Abdelwahab et al. [96] investigated the role of IL-6 and IL-6 receptors in the cytotoxic effects of zerumbone in ovarian and cervical cancer cell lines (Caov-3 and HeLa, respectively) using MTT assay. The studies suggested that zerumbone-induced cell death by stimulating apoptosis was found to be better than cisplatin, with significantly higher percentage of apoptotic cells in zerumbone’s treated cancer cells as compared with cisplatin as analyzed by flow cytometry. The study concluded that zerumbone inhibited cancer cell growth through the induction of apoptosis, arrested cells at the G2/M phase, and inhibited the secretion levels of IL-6 in both cancer cells. Thus, zerumbone was found to be a useful chemotherapeutic agent for treating both cervical and ovarian cancers in future.

Also, Liping et al. [97] studied sodium selenite to induce the apoptosis of cancer cells. MTT assay and morphological observation were used to study HeLa cells to get appropriate selenite concentrations for proteomic study. The study showed that selenite at concentrations larger than 10 μmol/l significantly inhibited the viability of HeLa cells. The results indicate that selenite induced the apoptosis of HeLa cells via ROS-mediated mitochondrial suppression, which could be used in cervical cancer treatment.

### 5.6. Dendrimers

Dendrimers are spherical, highly symmetric, and greatly branched macromolecules with a well-defined structure, surface charge, and molecular size that display a high degree of monodispersity [98]. Their structure allows the attachment and presentation of antigen molecules at their periphery, producing them as predominantly multifunctional. Drugs can be loaded into cavities in their cores by chemical linkages, hydrophobic interactions, hydrogen bonds, or conjugation to the polymer scaffold [99]. Polyacrylate star polymer conjugated to HPV E7 protein has been used to overcome the poor immunogenicity of peptide-based vaccines against cervical cancer. It was shown that these conjugates alone and after a single immunization were able to reduce tumor growth and remove E7-expressing TC-1 tumors in mice [100].

In a study by Mekuria et al. [101], doxorubicin-loaded dendrimers decorated with IL-6 antibody exhibited higher cellular internalization, lower IC_50_ value, higher drug loading, faster drug release rate, and more cytotoxicity compared with the RGD (arginyl-glycyl-aspartic acid) peptide-conjugated one in HeLa cells. This could probably be because of the higher multivalent ligand density on the surface of the IL6-conjugated dendrimers, which cause better drug delivery through receptor-mediated endocytosis.

### 5.7. Dendrosomes

Dendrosomes are novel vesicular, supramolecular, spherical entities in which the dendrimer–nucleic acid complex is encapsulated within a lipophilic shell. They have negligible hemolytic toxicity and higher transfection efficiency, and are better tolerated *in vivo* than are dendrimers. The word ‘dendrosome’ came from the Greek word ‘Dendron’ indicating tree and ‘some’ indicating vesicles. Thus, dendrosomes are vesicular structures composed of dendrimers [102,103]. In a study, Dutta et al. [104] explored the potential of dendrosomes for siRNA delivery to targeting E6 and E7 proteins of cervical cancer cells *in vitro*. The optimized N/P ratios were used in formulating complexes between dendrimers and siRNA targeting green fluorescence protein (siGFP). The formulation 4D100 (dendrimer–siRNA complex) showed the highest GFP knockdown and was encapsulated into dendrosomes in order to mask its toxic effects to cells. The GFP knockdown efficiency of DF3 (dendrosome) was identical with 4D100, but DF3 was non-toxic to the cells. Formulation (DF3) containing siRNA against E6 and E7 was found to knock down the target genes efficiently, as compared with the other formulations. The results indicated that dendrosomes hold potential for siRNA delivery to the target site.

## 6. Localized drug delivery systems

There are several benefits of localized drugs delivery to the cervix as compared with systemic delivery, such as prevention of systemic chemotherapeutic drug circulation resulting in less drug loss, decreased side effects, and delivery of a high dose of the active agent in the cervix, which in turn improves the efficacy of the treatment (Figure 5) [105]. The treatment of metastatic cervical tumors using localized drug carriers may be incompetent where the disease is spread in distant organs and demonstrates the necessity of a more systemic approach. However, the latest reports indicate that less than 20% of the cases appear to be with distant metastasis [106], which highlights the benefit of localized drug carriers for the treatment of cervical cancer.

As the cervix is easily reachable through the vagina, direct drug delivery is probable [107]. Currently, a variety of drugs in different forms, such as gels, rings, fibers, and tablets, are delivered through the vagina for several purposes, such as contraception or the treatment of fungal, bacterial, and sexually transmitted infections [108–110]. Improving the total quality of patients’ lives, localized delivery supports patients recuperating more speedily and diminishes the number of hospital presentations and admissions, reducing global healthcare system prices [111].

### 6.1. Hydrogels

Hydrogels are comprised of hydrophilic polymers dispersed into water forming a polymeric mesh concealing drug molecules inside [112], and have a tendency to swell for release of drug outside these polymeric meshes for disintegration and dissolution. Vaginally applied gels are established and known therapeutics, containing drugs and active ingredients which can refurbish physiological pH, moisturize and lubricate, be a contraceptive or labor inducer, and/or have microbicide activity [113]. Additional to swellability, hydrogels encompass physical properties such as mechanical resistance, permeability, surface features, and biocompatibility that can be altered through structural transformations [114].

Perez et al. [115] formulated a pH and glutathion-responsive, PTX-loaded poly-*N*-isopropylacrylamide (NIPA), tert-butyl 2-acrylamidoethyl carbamate (2AAECM) and *N*-hydroxyethyl acrylamide (HEAA)-based nanohydrogel by a microemulsion polymerization method comprising *N*,*N*'-cystaminebisacrylamide (CBA) as crosslinker. The developed formulations were assessed for cytotoxicity and cellular uptake studies using MTT assay and Coumarin-6, respectively, against cancer cell lines (MCF-7, T47D, and HeLa). The results indicated that Coumarin-6-loaded nanogel was rapidly taken up (in 2 h) and accumulated intracellularly (after 48 h). The favorable results of the cytotoxicity study suggested the use of developed nanogels as a novel nanocarrier of anticancer agents.

Jaiswal et al. [116] developed a pH- and temperature (dual stimuli)-sensitive, Fe_3_O_4_ magnetic nanoparticle-incorporated poly-(*N*-isopropylacrylamide)-chitosan-based nanohydrogel. The formulation was then evaluated for its drug delivery and antineoplastic potential by means of *in vitro* drug release and cytotoxicity studies using doxorubicin as the model drug. The developed system showed good response to both stimuli in respect of doxorubicin release. Furthermore, exposure to the cervical cancer (HeLa) cell line with concurrent heating of hydrogel-entrapped nanoparticles by means of an AC magnetic field revealed a significant decrease of cancerous cells.

Also, Curry et al. [117] developed a hydrogel system containing polyacrylamide-based nanoparticles with covalently linked Brilliant Blue G dye matrix for photothermal therapy of cervical cancer. The developed system was found to be effective at inducing thermolysis of the cervical cancerous (HeLa) cell line and so is anticipated to be pertinent and valuable in cervical-cum-other cancer therapy.

Sami and Kumar [118] developed three cryogel formulations based on chitosan-gelatin, chitosan-agarose, and chitosan-agarose gelatin by incorporation of microparticles into a polymeric mesh to obtain a supermacroporous multifunctional scaffold and assessed for biocompatibility and adherence using mouse myoblasts (C2C12), mouse embryonic fibroblasts (NIH 3T3), and human lung cancer cells (NCI-H460). The effectiveness of a cryogel–microparticles (C-MP) complex in drug delivery and cancer chemotherapy was evaluated by doxorubicin-loaded scaffold exposed to three-dimensional growing human cervical cancer cells (HeLa); an auspicious antiproliferative effect ensued.

Furthermore, Nazli et al. [119] designed ‘arginine-glycine-aspartic acid-serine’ (RGDS) (series derived from fibronectin so as to get the biofunctional PEG layer on to nanoparticles) functionalized ‘magnetic iron oxide nanoparticles’ (MIONPs) coated with polyethylene glycol hydrogel (PEG) using a surface-initiated photopolymerization technique. These newly designed MIONPs with a biofunctional active surface revealed a 17-fold higher uptake in the HeLa cell line with greater viability and stability and therefore proved to be a novel transporter of antineoplastic agents for improved cancer therapy.

Bilensoy et al. [120] formulated 5-FU vaginal gel using the thermosensitive polymer Pluronic F127 together with alternative mucoadhesive polymers, e.g. hyaluronic acid, Carbopol 934, and hydroxypropyl methylcellulose, to achieve a better therapeutic efficacy and patient compliance for the treatment of HPV-induced cervical cancers. The drug was incorporated as its inclusion complex with 1:1 molar ratio with either β-cyclodextrin or hydroxypropyl-β-cyclodextrin to increase its aqueous solubility and to achieve the complete release of 5-FU from the gel. The complete drug release from gels was obtained with both complexes of β-CD and HP-β-CD. Cytotoxicity studies using HeLa cells showed that 1% 5-FU:CD complexes were equally effective as 1% free 5-FU, indicating better therapeutic efficacy with lower dose.

Chun et al. [121] synthesized a poly(organophosphazene)–PTX conjugate by a covalent ester linkage between PTX and carboxylic acid-terminated poly(organophosphazene). The aqueous solutions of these conjugates showed a temperature-based sol–gel transition. The *in vitro* antitumor activity against HeLa cell lines using MTT assay and *in vivo* antitumor activity studies on tumor-induced (xenografted) nude mice revealed that the polymer–PTX conjugate hydrogels inhibited tumor growth more effectively over a longer period of time than PTX and saline alone. This indicates that poly(organophosphazene)–PTX conjugate holds promise for use in clinical studies related to cervix cancer as single and/or combination therapies.

In another approach a combination of immunotherapy and chemotherapy was reconnoitered by the investigators to improve the efficiency of cancer treatment. Seo et al. [122] formulated a novel biodegradable chitosan hydrogel, a co-delivery system for immunoadjuvants, and antineoplastic agents for advanced cancer therapy. The anticancer drugs (doxorubicin, cyclophosphamide, and cisplatin) along with GMCSF were incorporated and investigated against TC-1 cervical cells (expressing tumor-specific antigen HPV-16 E7) of mice to assess tumor growth. The results showed remarkable tumor suppression in only drug-hydrogel-treated mice, which was attributed to tumor antigen-specific T-cell-mediated antitumor immunity.

Moreover, Collaud et al. [123] developed 5-aminolevulinic acid (5-ALA) ester HAL and incorporated thermosetting hydrogel of poloxamer 407 for improved photodynamic therapy against CIN. The prepared hydrogel remains in solution form at room temperature and transforms into gel at body temperature when instilled in the genital tract (sol–gel transition). The *in vivo* and *in vitro* evaluation of the said formulation also represented promising release and cervical delivery of ALA [123,124]. The aforementioned hydrogel based delivery systems are summarized in Table 4.

**Table 4.** Hydrogel-based delivery systems, including cell lines/animal models used for cervical cancer therapy.

S. No.	Drug/therapy/functional group	Type/Composition of gel	Cell lines /animal models/neoplasm	References
1.	Paclitaxel	Poly-*N*-isopropylacrylamide, tert-butyl 2-acrylamidoethyl carbamate and *N*-hydroxyethyl acrylamide	MCF7, T47D, and HeLa cells	[115]
2.	Doxorubicin	Fe_3_O_4_ magnetic nanoparticle-incorporated poly-(*N*-isopropylacrylamide)-chitosan-based nanohydrogels	HeLa cells	[116]
3.	Photothermal therapy	Hydrogel system containing polyacrylamide-based nanoparticles	HeLa cells	[117]
4.	Doxorubicin	Cryogel formulations based on chitosan-gelatin, chitosan-agarose and chitosan-agarose gelatin	HeLa cells	[118]
5.	Arginine-glycine-aspartic acid-serine	Magnetic iron oxide nanoparticles coated with polyethylene glycol hydrogel	HeLa cells	[119]
6.	5-fluorouracil	Pluronic F127 together with alternative mucoadhesive polymers, e.g. hyaluronic acid, Carbopol 934, and hydroxypropyl methylcellulose	HeLa cells	[120]
7.	Paclitaxel	Temperature-sensitive poly(organophosphazene)–PTX conjugate	HeLa cells and nude mice	[121]
8.	Doxorubicin, cyclophosphamide and cisplatin	Chitosan hydrogel	Mice containing TC-1 cervical cells	[122]
9.	5-aminolevulinic acid (5-ALA) ester hexylamionolevulinate	Poloxamer 407	Cervical intraepithelial neoplasia	[123,124]

### 6.2. Scaffolds/nanofibers

Scaffolds are polymeric material in the form of injects or implants, which are used to deliver drugs, genes, and cells into the body. There are different types of polymeric scaffold for cell/drug delivery, e.g. the typical three-dimensional porous matrix [125], a nanofibrous matrix, a thermosensitive sol–gel transition hydrogel [126], and a porous microsphere. A scaffold acts as a suitable substrate for attachment of cells, their proliferation, differentiated function, and cell migration. A scaffold can be used to achieve high drug loading and drug delivery to specific sites [127]. Biomaterials used for preparation of the scaffold may be natural polymers such as alginate, collagens, gelatin, proteins, fibrins, and albumin, or synthetic polymers such as polyglycolide and polyvinyl alcohol [128].

Nanofibers have significant potential in drug delivery, especially in local chemotherapy. Their prominent features for electrospinning used in drug delivery are cost-effectiveness, high loading capacity, ease of operation, high encapsulation efficiency, and simultaneous delivery of various therapies [129].

Recently, drug-loaded ultrafine fibers have been used in local chemotherapy of cervical cancers. Biodegradable polylactide fiber mats loaded with PTX showed strong inhibition of xenograft U14 cervical cancer [130]. At the same drug level, the *in vivo* trials of cisplatin-loaded biodegradable poly(ethylene oxide)/polylactide composite electrospun nanofibers demonstrated more antitumor efficacy with better systemic safety than the intravenous injection group [131], indicating the benefits of localized delivery over systematic delivery.

Keskar et al. [132] developed a cisplatin-incorporated poly(ethylene-co-vinyl acetate) (EVAc) device similar to those used for vaginal contraceptive delivery. The dissolution from the devices was found to be biphasic and a controlled release was observed from the system with an initial rapid release phase followed by a linear slower release phase. The *in vitro* activity of devices established the effectiveness against both HPV-positive and HPV-negative cervical cancer cell lines. The studies indicated that the delivery system was a good candidate for the treatment of cervical cancers.

### 6.3. Bioadhesive patches

Woolfson et al. [133] developed a 5-FU-containing novel bioadhesive cervical patch containing a drug-loaded bioadhesive film cast from a gel containing 2% (w/w) Carbopol® 981 plasticised with 1% (w/w) glycerine for the treatment of CIN. The drug release through human cervical tissue samples was observed over approximately 20 h. Drug release was found to be clearly tissue rather than device dependent. The drug release and bioadhesive characteristics of the 5-FU cervical patch indicated its suitability for further clinical investigation as a drug treatment for CIN. Similarly, Iwata et al. [134] formulated a new stick-type formulation of Brilliant Blue or bleomycin hydrochloride, hydroxypropylcellulose (HPC), and a carboxyvinyl polymer (Carbopol 934) by direct compression with the possibility of continuous release of drugs over 1 week, when applied topically, and suggest the possibility of once-a-week treatment of uterine cervical cancer. By increasing the weight of the outer layer or the amount of HPC in the outer layer, the drug release *in vitro* from the sticks was found to be delayed.

In another study, a 5-FU-containing patch was paired with cervical tissue samples for a 24-h period. After this exposure, the tissue concentration of 5-FU was found to be 100 times that of the determined cytotoxic drug concentration. The result displays that for areas of the cervical stroma where precancerous lesions can occur, the patch delivery system could provide clinically effective drug concentrations [135]. However, the turnover of the mucosal lining limits these patches [136].

### 6.4. Nanogels

Choi et al. [137] developed a super-expandable smart nanogel that experienced nano- to micro-scale volume transition in response to temperature change, which is used for thermally triggered death of cancer cells. To enhance further the extent of cell death, various targeting ligands decipherable by specific cells could also be conjugated on the surface for controlling specific intracellular delivery. The anticancer drugs such as doxorubicin could also be loaded within the hydrophobic interior of the collapsed nanogels at 37°C to increase the cell lethality. Such target-specific and thermally detonatable ‘smart nanobomb’ particles loaded with potent cytotoxic drugs could be engineered for more effective necrotic and apoptotic cell death [138,139].

## 7. Herbal drug delivery systems against cervical cancer

The potential of herbal drug delivery systems for the treatment of cervical cancer has been explored by several researchers. Curcumin liposomal nanoparticles synthesized using didecyldimethylammonium bromide, cholesterol, and non-ionic surfactant showed distinct cytotoxicity, higher anticancer efficiency, and apoptosis compared with free curcumin in HeLa cells [140]. Similarly, curcumin-loaded liposomes coated with bioadhesive polymers of natural (chitosan) and synthetic (carbopol) origin were prepared. An *i* *n vitro* model of vaginal mucus was fabricated for evaluating curcumin permeability in an environment emulating the vaginal atmosphere, optimized by altering the quantity of glycoproteins, which was compared with isolated bovine mucus. Bioadhesion strength was examined using isolated bovine mucosa. Bioadhesive polymers revealed considerably superior (*p* < 0.05) curcumin permeability through simulated and isolated bovine mucus compared with the controls. Polymer coating of liposomes led to an increase in their bioadhesiveness. Mucoadhesive liposomes can be considered as imminent novel drug delivery systems scheduled for vaginal administration of curcumin [141].

Bioflavonoid naringenin-loaded nanoparticles prepared by a nanoprecipitation technique revealed greater cytotoxicity, increased intracellular ROS level, lipid peroxidation status, and decreased GSH level as compared with free naringenin in human cervical cancer cells [142]. Also, Green leaf extract of *Podophyllum hexandrum*-encapsulated AgNPs was prepared and found to cause DNA damage and caspase-mediated cell death of human cervical cancer cell selectively [143]. Moreover, crystalline gold nanoparticles synthesized using *Podophyllum hexandrum* L. were found to exhibit an effective anticancer activity by inducing oxidative stress, cell cycle arrest, DNA damage, and activation of caspase cascade, leading to mitochondrial dysfunction eventually ensuing in apoptosis of cancer cells [144].

PLGA nanoparticles loaded with curcumin were designed to improve the solubility and stability of curcumin which were further conjugated with anti-P-glycoprotein (APgp) antibody and were found to be beneficial in cancer patients either as a multidrug resistance modulator or as an anticancer drug [145]. Magnetic human serum albumin nanospheres containing 20(s)-ginsenoside Rg3- (extracted from red ginseng) were prepared by a desolvation crosslinking method and demonstrated conspicuous inhibition of cell growth including effectively induced apoptosis when combined with hyperthermia therapy in HeLa cervical cancer cells [146]. Magnetic iron oxide nanoparticles synthesized using brown seaweed aqueous extract were found to have an apoptosis-induced cytotoxic effect on cervical cancer cells [147]. Moreover, Ignatova et al. [148] have prepared novel nanofibrous mats coated with crosslinked quaternized chitosan containing natural polyphenolic compound gossypol (GOS) for cervical cancer treatment by the electrospinning technique. The GOS release from mats was found to be diffusion controlled and these mats revealed elevated cytotoxicity against human cervical cancer (HeLa) cells. The antiproliferative efficacy is attributed to cell apoptosis stimulation by nanofibrous mats.

Punfa et al. [149] compared the anticancer activity and cellular uptake of APgp-conjugated curcumin-loaded PLGA nanoparticles (Cur-NPs-APgp) and curcumin-loaded PLGA nanoparticles (Cur-NPs) in multidrug-resistant cervical cancer cells, KB-V1 (higher expression of Pgp) and KB-3-1 (lower expression of Pgp) using fluorescence microscope and flow cytometry, respectively. Cytotoxicity of the formulations was determined using MTT assay. The specific binding and cellular uptake of Cur-NPs-APgp to KB-V1 cells was found to be significantly higher than that to KB-3-1 cells. The cytotoxicity of Cur-NPs-APgp in KB-V1 cells was also higher than those of Cur and Cur-NPs. The results showed that Cur-NPs-APgp were targeted to Pgp on the cell surface membrane of KB-V1 cells, thus enhancing the cell uptake and cytotoxicity of curcumin.

Lekha et al. [150] developed curcumin nanoparticles (nanocurcumin) using two PLGA combinations, 50:50 and 75:25 ratios. Cellular uptake studies in HeLa cells exhibited enhanced intracellular fluorescence with nanocurcumin when compared with free curcumin. Antiproliferative studies using MTT assay, Annexin V/propidium iodide staining, poly(ADP-ribose) polymerase cleavage, and down-regulation of clonogenic potential of HeLa cells proved that the nanocurcumin 50:50 had better antitumor activiy. The results indicate that the enhanced aqueous solubility increased the anticancer efficacy of nanocurcumin. In another study by Altunbas et al. [151], an *in situ* self-assembling peptide hydrogel of curcumin (a hydrophobic polyphenol anticancer agent) was formulated for effective localized delivery over an extended period of time. The hydrogel encapsulation did not change the bioactivity of curcumin when assessed against the medulloblastoma cell line and its applicability was confirmed in treating other neoplastic conditions, including CIN.

Moreover, novel hybrid nanogels of curcumin were formulated by Wu et al. [152] for intracellular delivery. In this approach, Ag/Au bimetallic nanoparticles were first coated with a gel layer of hydrophobic polystyrene forming an inner core, which was further coated by a thin hydrophilic non-linear PEG-based gel comprising the outer core. This hybrid approach provides a distinctive advantage of assimilating functional building blocks for curcumin delivery and photothermal therapy at once, eventually improving therapeutic efficiency. The inner Ag/Au core of the system not only allows monitoring and imaging at the cellular level (by emitting fluorescence) but also provides a means of photothermal translation (by absorption in the near-infrared). Further, higher curcumin loading was observed with an inner hydrophobic core, while an outer thermoresponsive PEG core permits activation of curcumin release by means of exogenous near-infrared irradiation or surrounding temperature variation, and the formulation was claimed to be applicable in cervical and other cancer therapies.

PEG crosslinked acrylic hydrogel entrapping curcumin was prepared by Deepa et al. [153] using an inverse emulsion polymerization technique. The formulation showed sustained *i* *n vitro* curcumin release as the degree of crosslinking increased. The developed system was found to be favorable for pH-sensitive controlled delivery of curcumin and for cytotoxicity against human cervical cancer cell lines. Furthermore, Gonçalves et al. [154,155] prepared a self-assembled nanogel obtained from hydrophobically modified dextrin as an effective curcumin nanocarrier. The *in vitro* release studies indicated the dextrin nanogel as a suitable carrier for the effective controlled release of curcumin. The cytotoxic effect of curcumin on HeLa cell lines was not compromised when incorporated into nanogel. Thus, dextrin nanogel could be considered as a suitable drug delivery system for curcumin for the treatment of human cancers.

Vaginal films are promising delivery systems that could achieve better patient compliance and therapeutic efficacy. Curcumin-hydroxypropyl cyclodextrin complex vaginal films were shown to be retained in the vaginal mucosa for up to 6 h, making it a potentially effective therapy for HPV-induced cervical cancer [156]. Also, Li et al. [157] evaluated the cytotoxicity and apoptosis induction effects of a novel lipid-soluble extract (PE) from *Pinellia pedatisecta* Schott on CaSki, HeLa, and HBL-100 cells. In particular, the effect of PE on HPV E6 gene expression was tested, and the mechanism of its apoptosis induction effect was also studied. Cell viability was measured by the MTT assay. PE inhibited the growth of CaSki and HeLa cells in a time- and dose-dependent manner, but it had no obvious inhibiting effect on HBL-100 cells except at a relatively high dose (500 μg/ml). Thus, PE acted as a tumor suppressor by inducing apoptosis in human cervical cancer cells but it had little side effect on normal cells. HPV E6 may be the key target of its action.

The anticancer activity of AgNPs synthesized using *Taxus baccata* extracts was studied on Caov-4 and HeLa cancer cell lines as well as normal human fibroblast cells. Microscopic studies showed significant morphological changes of cancer cells following the exposure to the AgNPs while MTT assay revealed dose, time and cell line-dependent cytotoxicity. The toxic effect of AgNPs on Caov-4 cells was considerably higher than HeLa cells as more than 98% mortality was obtained after 72 h incubation of Caov-4 cells with 5 and 20 µg/ml AgNPs encapsulated by aqueous and ethanolic extract, respectively [158].

## 8. Brief overview of photothermal/radiation/gene therapy

Photothermal therapy is comprised of infrared-wavelength-range electromagnetic radiation for treating a number of medical circumstances, including cancer, which is less harmful to non-cancerous cells and tissues and also less energetic [159]. Gene therapy approaches involve gene manipulation on broad biological processes accountable for the spreading of diseases, hence making them a potential approach for the treatment of diverse cancers. Radiotherapy is a treatment using high-energy X-rays and is generally given by linear accelerator machines. Radiotherapy causes changes in normal cells as well as cancer cells, but cancer cells are more sensitive to radiotherapy and so more of them are killed. Normal cells are better able to repair themselves and hence the damage to normal cells is mainly transitory [160].

The role of the above-mentioned therapies in the treatment of cervical cancer is briefly summarized in Table 5

**Table 5.** Brief overview of radiotherapy, photothermal therapy, and gene/recombinant protein therapy.

Types of therapy	Carrier system/formulation	Targeting ligand	Result	Reference
Radiotherapy	Bovine serum albumin nanoparticles as carriers of organic selenocompound,	Folate as a targeting ligand	Apoptosis as well as cell cycle arrest of cervical cancer cells	[161]
	multifunctional carbon nanohorn complexes	Ferromagnetic nanoparticles	Significant increase in the photothermal effect of complex due to laser irradiation leading to increased cell death in cervical cancer	[162]
	PEGylated glucose gold nanoparticles	Polyethylene glycol ligands	Better half-life, excellent *in vivo* stability, enhanced tumor targeting and radiotherapeutic effects	[163]
Photothermal therapy	Doxorubicin superparamagnetic iron oxide nanoparticles	Folic acid	Increased cellular uptake and therapeutic efficiency	[164]
	Doxorubicin-loaded graphene oxide	Polyethylene glycol	Excellent biocompatibility, high drug loading and photothermal conversion efficiency	[165]
	Doxorubicin-loaded silica graphene-serum nanoparticles		Higher photothermal conversion efficiency, and stability, increased storage and release capacity for doxorubicin, led to the enhanced cytotoxicity	[166]
	Hollow gold nanospheres of p65 siRNA and irinotecan	siRNA recognizing NF-κB p65	Significant increase in tumor apoptosis and growth delay	[167]
	Gold colloidal nanoparticles		Increased cytotoxic effect on epithelial cancer cells	[168]
Gene and recombinant protein therapy	DNA vaccine containing HPV oncoproteins and herpes virus gD protein	–	Enhanced stimulation of antigen-specific cytotoxic CD8(+) T-cell responses, full therapeutic antitumor effects	[169]
	Novel vaccine carrier composed of HPV-16 E7 and ricin toxin (RTB) lectin subunit fusion	–	Enhanced activity with respect to E7 immunization alone	[170]
	VLP-E7 vaccine	–	A potent and long-lasting immune response	[171]
	Recombinant IkBα-loaded curcumin nanoparticles	–	Enhanced therapeutic efficacy of recombinant IkBα protein led to apoptotic cell death	[172]
	Folate poly(ethylene glycol)-b-poly(D,L-lactide) gene-loaded nanoparticles for gene loading	–	Increase in nuclear uptake of genetic material	[173]
	CRISPR/Cas9 as targeting promoter of HPV 16 E6/E7 transcripts	–	Significant reduction in proliferation of cervical cancer cells *in vitro* and *in vivo*	[174]
	Phosphorothioate oligodeoxynucleotides (PT-oligos)	–	Significant antiproliferative activity against oncoproteins of HPV cancerous cells	[175]
	Recombinant vaccinia vaccine (TA-HPV) expressing E6 and E7 for HPV-16 and 18	–	Excellent efficacy in Phase II study in women’s group with HPV-positive vulval and vaginal intraepithelial neoplasia	[176]
	Recombinant human TNF-related apoptosis-inducing ligand (rhTRAIL)		Effective in cervical cancer	[177]
	TPGS-b-(PCL-ran-PGA) nanoparticle	Polyethyleneimine (PEI) carrying TRAIL and/or endostatin genes	Enhanced cytotoxicity towards HeLa cells	[178]

## 9. Conclusion

Drug resistance remains an important obstacle to better outcomes in the treatment of cancer by conventional drug therapy. Resistance provided in the case of conventional therapy can be overcome by the newer approach of drug delivery systems. Colloidal drug carriers such as liposomes and nanoparticles are able to modify the distribution of an associated drug substance which can overcome the drug resistance. Alternatively, drug delivery approaches using nano-colloidal carriers can in principle target drugs to tissue, cellular, and subcellular target sites. By increasing bioavailability of drugs at sites of action, these approaches may provide therapeutic advantages, including enhanced efficacy against resistance in the case of cervical cancer. These approaches seek to overcome drug resistance by more efficient delivery to target cells and in some cases by concomitant avoidance or inhibition of drug efflux mechanisms. These drug delivery systems in combination with localized delivery systems, photothermal, radiation, and gene therapy are found to be reasonably effective and could display a new way to eliminate cervical cancer completely.
